# Immune-Mediated Hypophysitis: An Updated Review

**DOI:** 10.3390/jcm15093313

**Published:** 2026-04-27

**Authors:** Pedro Iglesias

**Affiliations:** 1 Department of Endocrinology and Nutrition, Hospital Universitario Puerta de Hierro Majadahonda, 28222 Madrid, Spain; piglo65@gmail.com; 2 Instituto de Investigación Sanitaria Puerta de Hierro Segovia de Arana, 28222 Madrid, Spain

**Keywords:** immune-mediated hypophysitis, hypophysitis, IgG4-related hypophysitis, immune checkpoint inhibitor-induced hypophysitis, lymphocytic hypophysitis, magnetic resonance imaging, paraneoplastic immune-mediated hypophysitis, pituitary hormone deficiencies

## Abstract

**Background:** Immune-mediated hypophysitis comprises a heterogeneous group of inflammatory pituitary disorders, including primary lymphocytic hypophysitis, immune checkpoint inhibitor (ICI)-induced hypophysitis, IgG4-related hypophysitis, and paraneoplastic autoimmune hypophysitis. Although these entities share immune-mediated mechanisms, they differ substantially in clinical presentation, imaging features, and therapeutic implications. **Methods:** This narrative review synthesizes current evidence on the pathophysiology, clinical manifestations, radiological characteristics, diagnostic approach, and management of immune-mediated hypophysitis, with particular emphasis on etiological heterogeneity. **Results:** Hypopituitarism—particularly ACTH deficiency—is the most frequent and clinically relevant manifestation, as secondary adrenal insufficiency may be life-threatening if not promptly recognized and treated. It is often accompanied by headache, arginine vasopressin deficiency, or mass effect depending on the subtype. Magnetic resonance imaging typically shows symmetrical pituitary enlargement and stalk thickening in inflammatory forms, although findings vary according to etiology and may be minimal in certain subtypes such as PD-1/PD-L1 inhibitor-associated hypophysitis. Distinct clinical phenotypes are observed across subtypes, particularly in ICI-induced hypophysitis and IgG4-related disease. Diagnosis relies on the integration of endocrine, radiological, and clinical features, supported by clinicoradiological scoring systems in selected cases. Management is primarily based on prompt hormone replacement, with selective use of glucocorticoids or immunosuppressive therapies depending on disease severity and underlying etiology. **Conclusions:** Immune-mediated hypophysitis represents a clinically relevant and increasingly recognized spectrum of disorders requiring a multidisciplinary and etiology-specific approach. Early recognition is essential to prevent life-threatening endocrine complications. Advances in the understanding of immunopathogenic mechanisms and the identification of reliable biomarkers may enable earlier diagnosis and more personalized therapeutic strategies.

## 1. Introduction

**Figure 1:**
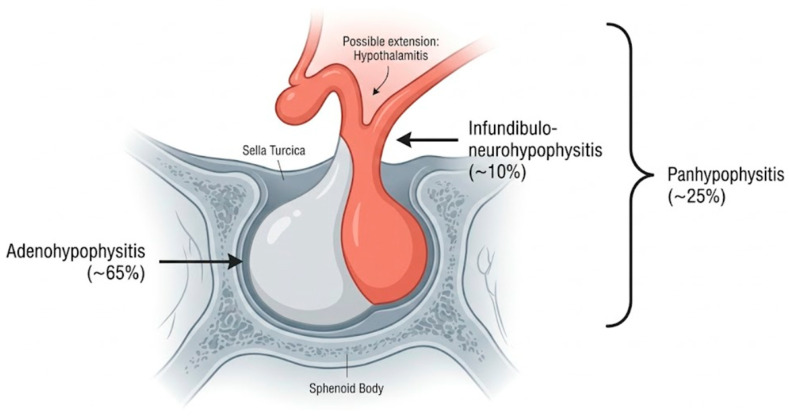
Anatomical patterns of hypophysitis based on the distribution of inflammatory involvement.

Hypophysitis is defined as an inflammatory process affecting the pituitary gland and/or infundibulum, which may compromise the adenohypophysis, neurohypophysis, or both structures. It encompasses a heterogeneous group of entities that can be classified according to their etiology into primary (idiopathic or autoimmune) and secondary forms (associated with systemic diseases, infections, neoplasms, or drugs such as immunotherapy), and according to their anatomical location into adenohypophysitis, neurohypophysitis, infundibuloneurohypophysitis, or panhypophysitis [1,2,3,4,5] (Figure 1). Among these, immune-mediated hypophysitis represents a clinically and pathophysiologically distinct subset that has gained increasing attention in recent years.

Immune-mediated forms—including primary lymphocytic hypophysitis (LH), immune checkpoint inhibitor (ICI)-induced hypophysitis, IgG4-related hypophysitis, and paraneoplastic autoimmune variants—share common immunological mechanisms but differ in epidemiology, clinical presentation, and management. Their recognition is clinically relevant because they often require a tailored diagnostic approach and may have different therapeutic implications compared with other inflammatory or neoplastic sellar lesions.

The clinical presentation of hypophysitis typically includes symptoms of hormonal dysfunction (hypopituitarism and/or arginine vasopressin deficiency (AVP-D) syndrome, formerly known as central diabetes insipidus) and/or symptoms related to mass effect (headache and/or visual disturbances). Secondary adrenal insufficiency due to ACTH deficiency represents the most critical endocrine complication, as delayed recognition and treatment may be life-threatening. In addition, involvement of the posterior pituitary may lead to AVP-D, which also requires prompt recognition and appropriate management. Diagnosis relies on the integration of clinical, hormonal, and imaging findings, with magnetic resonance imaging being the imaging modality of choice to detect thickening of the pituitary stalk and homogeneous enlargement of the gland. In selected cases with diagnostic uncertainty, a transsphenoidal biopsy may be required to confirm inflammation and rule out alternative etiologies, including germ cell tumors, Langerhans cell histiocytosis, metastatic lesions, lymphoma, or other infiltrative and infectious processes [1,3,4,5,6].

The management of hypophysitis is mainly based on hormone replacement, prioritizing the correction of corticotropic (ACTH) insufficiency and other pituitary deficits [3]. High-dose immunosuppressive doses of glucocorticoids are reserved for cases with mass effect or specific forms such as IgG4-related hypophysitis. In checkpoint inhibitor-induced hypophysitis, replacement therapy and conservative management are usually chosen, and oncologic therapy can often be continued under close monitoring [7,8,9]. Surgery is limited to situations of diagnostic uncertainty or progressive visual impairment [5,10].

## 2. Methodology of the Review

This narrative review was conducted following a structured literature search strategy to identify relevant evidence on immune-mediated hypophysitis. The PubMed/MEDLINE database was systematically searched for articles published between January 1990 and December 2025 using combinations of the following keywords and MeSH terms: hypophysitis, immune-mediated hypophysitis, lymphocytic hypophysitis, immune checkpoint inhibitor-induced hypophysitis, IgG4-related hypophysitis, paraneoplastic hypophysitis, and pituitary autoimmunity.

Only articles published in English were considered. Eligible publications included original clinical studies, observational cohorts, translational research, systematic reviews, and consensus statements addressing the pathophysiology, clinical presentation, diagnosis, imaging features, or management of immune-mediated hypophysitis. Case reports and studies with insufficient methodological detail were considered only when providing relevant insights into rare subtypes or emerging mechanisms.

Titles and abstracts were screened to identify relevant publications, followed by full-text evaluation when appropriate. Additional studies were identified through manual screening of reference lists from selected articles. The retrieved literature was qualitatively synthesized, focusing on immunopathogenic mechanisms, clinical manifestations, radiological characteristics, diagnostic strategies, and therapeutic approaches across the main subtypes of immune-mediated hypophysitis. Emphasis was placed on studies with clear methodological rigor and clinical relevance.

## 3. Rationale for the Review

Immune-mediated hypophysitis has gained increasing clinical relevance over the past decade, largely driven by the widespread use of cancer immunotherapy, improved recognition of IgG4-related disease, and advances in diagnostic techniques. Despite these developments, significant gaps remain in the understanding of its pathophysiological mechanisms, the standardization of diagnostic criteria, and the optimization of etiology-specific therapeutic strategies. The marked clinical, radiological, and immunological heterogeneity of these entities continues to hinder early diagnosis and uniform management in routine practice. In this context, an updated and integrative review is warranted to synthesize current knowledge on the anatomical and immunopathogenic bases, emerging biomarkers, and clinical implications of the different forms of immune-mediated hypophysitis, with the aim of improving recognition, risk stratification, and patient care.

## 4. Pituitary Gland and Immunity

The pituitary gland is currently recognized as a key organ within the neuroendocrine–immune network, characterized by complex bidirectional communication with the immune system. Beyond its classic endocrine function, experimental and clinical evidence shows that the pituitary gland can simultaneously act as a sensor of inflammatory signals, a modulator of the immune response, and a target organ for autoimmune phenomena, providing the conceptual framework for understanding immune-mediated hypophysitis [11,12,13] (Table 1).

During systemic inflammatory processes, adenohypophyseal cells respond to proinflammatory cytokines—especially IL-1β, IL-6, and TNF-α—with corticotropes being particularly sensitive through increased ACTH secretion and activation of the hypothalamic–pituitary–adrenal (HPA) axis, the main homeostatic pathway for inflammation control [12,14,17,18,20]. This axis induces the production of cortisol, which exerts pleiotropic anti-inflammatory effects on lymphocytes and macrophages. Likewise, pituitary cells can release POMC-derived peptides with immunomodulatory activity and express chemokines that facilitate the recruitment of immune cells, supporting a dynamic role for the pituitary gland in the regulation of the systemic inflammatory response [17,18,19].

At the same time, the existence of an intra-pituitary immune microenvironment composed of resident macrophages, dendritic cells, and local production of cytokines and chemokines has been demonstrated [13,16]. The unique fenestrated vascularization of the pituitary gland promotes exposure to systemic mediators and cell trafficking, which could contribute to its susceptibility to inflammatory and autoimmune processes [11,15].

A paradigmatic clinical model of induced autoimmunity is hypophysitis associated with ICI. It has been demonstrated that pituitary cells express CTLA-4, which allows the binding of ipilimumab and local complement activation, generating a type II hypersensitivity mechanism with tissue damage [9,23,24,28]. IgG4-related hypophysitis falls within the spectrum of IgG4-related systemic disease and is defined by dense infiltration of IgG4 plasma cells and characteristic fibrosis [25,26].

In addition, paraneoplastic autoimmune phenomena targeting the pituitary gland have been described, probably mediated by antigenic mimicry between tumor antigens and pituitary proteins; however, the clinical relevance of this mechanism remains uncertain and requires further confirmation [27,29].

Overall, the available evidence supports the idea that the pituitary gland acts as an integrative node for systemic and local immunoregulation. The ability to respond to cytokines, modulate inflammation through the HPA axis, harbor resident immunity, and become an autoimmune target constitutes the pathophysiological basis on which immune-mediated hypophysitis develops and explains its clinical, radiological, and hormonal manifestations.

## 5. Epidemiology and Emerging Trends in Immune-Mediated Hypophysitis

The epidemiology of immune-mediated hypophysitis is heterogeneous because the denominator differs across subtypes: primary autoimmune forms are rare at the population level, whereas immune checkpoint inhibitor (ICI)-related hypophysitis is more appropriately expressed as a proportion of exposed oncology cohorts. Moreover, variability in case ascertainment (radiological hypophysitis vs. isolated ACTH deficiency; systematic screening vs. clinically driven testing) can substantially influence reported rates [30]. The main epidemiological estimates for the principal immune-mediated hypophysitis subtypes are summarized in Table 2.

Primary LH remains an uncommon disorder, accounting for a very small proportion of pituitary lesions. Although classic epidemiological estimates have been reported, these figures are likely influenced by underdiagnosis and limited histological confirmation [30]. Data from large registries confirm the rarity of primary hypophysitis and the clear predominance of the lymphocytic subtype within this group [31]. Historically, the disease predominantly affected young women, particularly in relation to pregnancy or the postpartum period; however, more recent cohorts suggest greater demographic heterogeneity, indicating that earlier descriptions may have been influenced by selection bias [10,32]. Overall, autoimmune (predominantly lymphocytic) hypophysitis represents a minority of pituitary masses, highlighting its marked rarity in comparison with pituitary adenomas [30].

Although hypophysitis has long been considered rare, its apparent incidence has increased in recent years, reflecting both improved recognition and true epidemiological changes related to the widespread use of cancer immunotherapy [10]. Currently, ICI-induced hypophysitis has become the most common cause of secondary hypophysitis in many tertiary centers and shows a distinct epidemiological profile, with a higher incidence in older men and in patients with malignancies treated with immunotherapy [7,8,9]. The risk varies according to the class of immunotherapy, being higher with CTLA-4 inhibitors and combination regimens, and lower with PD-1/PD-L1 inhibitors [33,34].

IgG4-related hypophysitis represents a small but likely underrecognized subset because of its clinical and radiological overlap with other sellar inflammatory conditions [25,26]. Available data suggest that it may account for a non-negligible proportion of cases in selected cohorts and that some cases historically classified as primary hypophysitis may correspond to IgG4-related disease [35].

Paraneoplastic immune-mediated hypophysitis, including anti-PIT-1 syndrome, remains exceptionally rare and lacks reliable population estimates. Current evidence is largely limited to small case series, underscoring the considerable uncertainty regarding its true incidence and the likelihood of underrecognition [27,36].

**Table 2:** Epidemiology of immune-mediated hypophysitis subtypes.

Subtype	Incidence/Prevalence (with Denominator)	Evidence Context (Denominator)	Key Limitations	References
**Lymphocytic hypophysitis**	Prevalence ≈ 5 per million; annual incidence ≈ 1 per 7–9 million	Population-level clinical estimate	Likely underdiagnosis; limited histological confirmation	[30]
	4/1293 (0.31%) within pituitary disorders cohort	Referral cohort of pituitary diseases	Referral bias; non-population denominator	[37]
	<1% of pituitary masses; ≈2% of nonfunctioning macro-lesions	Surgical/clinical pituitary series	Depends on case ascertainment and surgical selection	[30]
**ICI-induced hypophysitis**	37/954 (3.9%) HPA dysfunction attributed to ICI	ICI-treated oncology cohort	May include isolated ACTH deficiency without radiological hypophysitis	[38]
	17/154 (11.0%) with ipilimumab in metastatic melanoma	Anti-CTLA-4 exposure cohort	Tumor-specific population; surveillance intensity	[28]
	6/25 (24%) ipilimumab vs. 10/167 (6.0%) anti-PD-1	Prospective oncology cohort	Small CTLA-4 sample; definition-driven rates	[39]
	Anti-CTLA-4 ≈ 3–6%; PD-1/PD-L1 ≈ 0.3–1%; combination ≈ 6.4% (up to ~19%)	Meta-analyses and pooled series	Heterogeneity in definitions and monitoring	[8,9,33]
**IgG4-related hypophysitis**	7/170 (4.1%) among hypopituitarism/central DI	Systematic endocrine screening cohort	Enriched denominator; not population-based	[40]
	12/29 (41.4%) of histologically confirmed primary hypophysitis	Neuropathology reclassification	Strong selection bias (biopsied cases)	[35]
**Paraneoplastic autoimmune hypophysitis**	Incidence not estimable; 9 cases reported	Case-based literature	No denominator; publication bias	[36]
	Conceptual entity supported by small series	Narrative/review evidence	Extreme rarity; likely underrecognition	[27]

Abbreviations: ACTH, adrenocorticotropic hormone; CTLA-4, cytotoxic T-lymphocyte-associated antigen 4; HPA, hypothalamic–pituitary–adrenal axis; ICI, immune checkpoint inhibitor; IgG4, immunoglobulin G4; PD-1, programmed cell death protein 1; PD-L1, programmed death ligand 1.

## 6. Etiopathogenic Mechanisms of Immune-Mediated Hypophysitis

**Figure 2:**
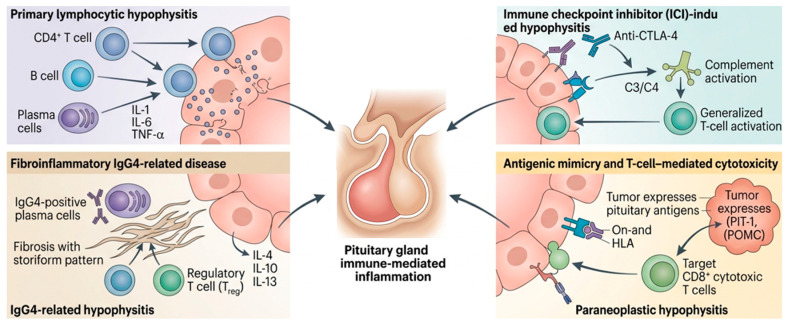
Pathogenic mechanisms of immune-mediated hypophysitis. Schematic representation of the main immunopathogenic pathways underlying immune-mediated hypophysitis. Primary lymphocytic hypophysitis is characterized by organ-specific autoimmunity driven by CD4^+^ T cells, B cells, and plasma cells, with proinflammatory cytokine release (IL-1, IL-6, TNF-α). Immune checkpoint inhibitor (ICI)-induced hypophysitis results from immune disinhibition, particularly with anti-CTLA-4 therapies, involving complement activation and T-cell–mediated inflammation. IgG4-related hypophysitis represents a fibroinflammatory condition characterized by IgG4-positive plasma cells, regulatory T-cell responses, and cytokine-driven fibrosis (IL-4, IL-10, IL-13). Paraneoplastic hypophysitis arises from antigenic mimicry, whereby tumor expression of pituitary antigens (e.g., PIT-1, POMC) triggers HLA-restricted activation of cytotoxic CD8^+^ T cells targeting pituitary cells. All pathways converge in pituitary inflammation and varying degrees of hormonal deficiency.

Immune-mediated hypophysitis encompasses a heterogeneous group of disorders characterized by immune-driven inflammation of the pituitary gland. Although these entities share common autoimmune features, their pathogenic mechanisms differ according to the underlying trigger and the dominant immunological pathways involved (Figure 2).

The main immunopathogenic mechanisms involved in the different forms of immune-mediated hypophysitis are summarized in Table 3, highlighting the distinct immune triggers, dominant cellular pathways, and histopathological features associated with LH, (ICI)-induced hypophysitis, IgG4-related hypophysitis, and paraneoplastic immune-mediated hypophysitis.

LH is an organ-specific autoimmune disease characterized by lymphoplasmacytic infiltration and progressive destruction of pituitary tissue. Its pathogenesis is multifactorial and involves the interaction of cellular and humoral immunity against a background of genetic susceptibility and hormonal factors [41]. The central finding is a predominant infiltration of CD4^+^ T lymphocytes, accompanied by B lymphocytes and plasma cells, with a Th1/Th17 immune profile that promotes inflammation and tissue damage [21,41]. The presence of anti-pituitary autoantibodies supports the autoimmune nature, although their direct pathogenic role is uncertain [1,22]. The association with HLA haplotypes, concomitant autoimmune diseases, and predominance in young women—especially in relation to pregnancy or the postpartum period—suggests genetic and hormonal modulation of the process [1,41]. Initially, inflammation causes glandular enlargement and hormonal dysfunction, but progression can lead to fibrosis and permanent pituitary deficiency.

Immune checkpoint inhibitor (ICI)-induced hypophysitis is a form of secondary autoimmunity triggered by the pharmacological disinhibition of the antitumor immune response. Its mechanism differs from that of classic LH and depends largely on the type of drug involved. In anti-CTLA-4-associated hypophysitis (especially ipilimumab), the best-characterized mechanism is an antibody-mediated autoimmune reaction. It has been shown that pituitary cells express CTLA-4, allowing direct binding of the drug and local complement activation, generating a type II cytotoxicity process with inflammatory infiltration and tissue damage. This model explains the higher incidence and more florid presentation observed with CTLA-4 inhibitors compared to anti-PD-1/PD-L1 inhibitors [23,33,42]. From a cellular perspective, the disinhibition of T lymphocytes promotes the expansion of autoreactive clones and the loss of peripheral tolerance. The pituitary infiltrate is usually less organized than in the primary lymphocytic form and less prone to fibrosis, suggesting a more acute and pharmacologically induced immune process. In addition, the presence of anti-pituitary autoantibodies induced or amplified after exposure to ICI has been described, which could act as biomarkers of susceptibility [43]. There are relevant mechanistic differences between therapeutic classes. CTLA-4 inhibitors are associated with true inflammatory hypophysitis with glandular enlargement, while PD-1/PD-L1 inhibitors are more frequently associated with isolated ACTH deficiency without marked radiological inflammation, suggesting partially distinct and possibly more selective immune mechanisms on corticotropes [24,45].

IgG4-related hypophysitis represents a distinct fibroinflammatory form of immune-mediated pituitary disease that belongs to the broader spectrum of IgG4-related disease (IgG4-RD), a systemic immune-mediated condition characterized by tumefactive lesions, dense lymphoplasmacytic infiltration rich in IgG4-positive plasma cells, and varying degrees of storiform fibrosis [25,26,35,40,46,47,48]. The pathogenesis is incompletely understood but appears to involve complex interactions between innate and adaptive immune mechanisms. A key immunological feature is the expansion of IgG4-secreting plasma cells driven by activated B cells and T helper cells, particularly a Th2- and regulatory T cell (Treg)-dominant immune environment with increased production of cytokines such as IL-4, IL-10, and IL-13 [25]. These cytokines promote class switching toward IgG4 production and contribute to the development of chronic fibroinflammatory tissue remodeling. In contrast to classic LH, the inflammatory infiltrate in IgG4-related hypophysitis is typically accompanied by prominent fibrosis and, in some cases, obliterative phlebitis, histopathological features characteristic of IgG4-related disease in other organs [25,26,35,46,49]. The disease may occur as part of multisystem IgG4-related disease involving organs such as the pancreas, salivary glands, retroperitoneum, or lymph nodes, although isolated pituitary involvement has also been described [46,47]. From an immunopathogenic perspective, regulatory immune responses appear to play a central role; paradoxically, IgG4 antibodies themselves are considered relatively non-inflammatory and may represent a secondary phenomenon reflecting chronic immune activation rather than the primary pathogenic driver [48,50]. Genetic susceptibility, environmental triggers, and molecular mimicry have been proposed as potential contributors to disease initiation, although definitive mechanisms remain uncertain. The progressive fibroinflammatory process may lead to pituitary enlargement during the active phase, followed by structural remodeling and persistent hypopituitarism if untreated. Recognition of IgG4-related hypophysitis as a distinct immunological entity is clinically relevant, as it differs from other forms of immune-mediated hypophysitis in both pathophysiology and systemic associations and may respond particularly well to glucocorticoid therapy and B-cell-directed treatments in selected cases [25,26].

Paraneoplastic immune-mediated hypophysitis represents a rare but increasingly recognized form of secondary autoimmune pituitary inflammation, characterized by an immune response directed against antigens shared by tumor and pituitary tissues [27,29]. This entity is best understood within the emerging framework of “onco-immuno-endocrinology,” in which ectopic tumor antigen expression drives endocrine autoimmunity [51]. The central pathogenic mechanism relies on antigenic mimicry, whereby ectopic expression of pituitary-restricted antigens—such as the transcription factor PIT-1 or pro-opiomelanocortin (POMC)—enables HLA-dependent antigen presentation and subsequent breakdown of immune tolerance [27,52]. This process involves a coordinated sequence of events including ectopic antigen expression, activation of autoreactive cytotoxic T lymphocytes (CTLs), and immune-mediated destruction of specific pituitary cell lineages [52]. Available evidence indicates that this process is predominantly T cell-mediated. Tumor-driven antigen presentation promotes the expansion of antigen-specific CD8^+^ cytotoxic T lymphocytes together with CD4^+^ helper T-cell responses, which infiltrate the anterior pituitary and induce selective endocrine cell destruction [53]. Genetic susceptibility, particularly specific HLA haplotypes (e.g., HLA-A24:02, HLA-A02:06, DR53), may further modulate disease development [36].

Anti-PIT-1 hypophysitis represents the best-characterized model of this entity. The restricted expression of PIT-1 in somatotroph, lactotroph, and thyrotroph lineages explains the distinctive pattern of combined growth hormone (GH), prolactin, and thyroid-stimulating hormone (TSH) deficiency observed in affected patients [36,54]. Circulating anti-PIT-1 antibodies serve as disease markers, whereas PIT-1-reactive CTLs are considered the principal mediators of tissue damage [54]. Thymomas represent the most frequently associated tumors, and tumor removal has been shown to reduce both antibody titers and T-cell reactivity, supporting a causal relationship [54].

In addition to PIT-1-related disease, a second, less well-established form involves selective corticotroph dysfunction associated with POMC-expressing tumors, particularly neuroendocrine malignancies [52]. In this context, ectopic POMC expression may trigger autoreactive immune responses leading to isolated ACTH deficiency. Both anti-POMC antibodies and POMC-reactive CTLs have been described, although available evidence remains limited and primarily based on small case series [52].

A related and partially overlapping mechanism has been proposed in a subset of immune checkpoint inhibitor (ICI)-related hypophysitis, in which enhanced antitumor immunity may amplify autoreactive responses against shared tumor–pituitary antigens, particularly affecting corticotroph cells [8,55].

## 7. Clinical Presentation of Immune-Mediated Hypophysitis

### 7.1. Lymphocytic Hypophysitis

Primary LH is the prototypical form of primary immune-mediated hypophysitis and typically presents with varying degrees of anterior pituitary dysfunction and/or symptoms related to sellar mass effect. The clinical phenotype is variable and largely depends on the anatomical compartment involved (adenohypophysis, neurohypophysis, or both), the intensity of inflammation, and the timing of diagnosis [1,10,25,56].

From an endocrine perspective, hypopituitarism constitutes the most frequent clinical manifestation. Adrenocorticotropic hormone (ACTH) deficiency is usually the earliest and most clinically significant hormonal deficit, occurring in approximately 60–80% of patients at diagnosis, and is of particular clinical relevance because of the risk of life-threatening adrenal insufficiency. In terms of frequency, ACTH deficiency is followed by central hypothyroidism and hypogonadotropic hypogonadism, reported in approximately 40–70% and 40–60% of cases, respectively [10,56]. The reported prevalence of growth hormone (GH) deficiency in primary LH is approximately 22%, although it is likely to be underestimated in adults due to diagnostic limitations [57]. The clinical presentation may be nonspecific, including fatigue, weakness, hypotension, nausea, or hyponatremia, which can delay recognition [10].

Hyperprolactinemia due to stalk compression may be observed, although prolactin deficiency can occur in advanced destructive stages [58]. Hormonal deficits may evolve over time, and partial deficiencies at presentation can progress to panhypopituitarism if inflammation persists. When the posterior pituitary and/or infundibulum are involved, an AVP-D syndrome may develop, manifesting with polyuria and polidipsia [59]. AVP-D has been reported in up to 30–50% of patients with posterior involvement, and the coexistence of anterior hypopituitarism and AVP-D is considered highly suggestive of an inflammatory or infiltrative sellar process rather than a typical pituitary adenoma [60].

In addition to endocrine dysfunction, LH may present with local compressive symptoms during the inflammatory phase, when pituitary enlargement is most prominent. Headache is the most common local symptom, reported in approximately 50–80% of patients, and is often disproportionate to the size of the lesion observed on imaging [10,61]. Visual disturbances due to compression of the optic chiasm—such as bitemporal hemianopsia or decreased visual acuity—are less frequent but clinically relevant, occurring in roughly 10–30% of cases [1,10,61]. Rarely, lateral extension toward the cavernous sinus may produce diplopia or ophthalmoplegia.

The natural history of LH typically follows a biphasic course. An initial inflammatory stage characterized by lymphocytic infiltration and glandular enlargement may be followed by a chronic phase with fibrosis and progressive loss of pituitary tissue, resulting in permanent endocrine deficits in a substantial proportion of patients [62,63,64]. Radiologically, pituitary enlargement may decrease spontaneously over time or after glucocorticoid therapy, sometimes evolving toward pituitary atrophy or empty sella, although morphological improvement does not necessarily correspond to recovery of pituitary function [65,66].

### 7.2. Granulomatous Hypophysitis

Granulomatous hypophysitis is a rare form of primary hypophysitis characterized histologically by multinucleated giant cells, granuloma formation, and variable fibrosis. It represents the second most common subtype of primary hypophysitis and differs from lymphocytic hypophysitis in several clinical and immunological aspects [67,68]. Unlike the lymphocytic form, it shows no clear association with pregnancy and tends to affect both sexes more evenly [67].

Clinically, it typically presents with headache, hypopituitarism, and, less frequently, visual disturbances, with a relatively high prevalence of AVP-D reflecting posterior pituitary involvement [10,69]. From a pathophysiological perspective, it is considered an idiopathic inflammatory process once secondary causes—such as infections or systemic granulomatous diseases—have been excluded, and appears to involve a distinct immune mechanism characterized by cytotoxic T-cell and histiocytic infiltration [10,68].

Compared with lymphocytic hypophysitis, it may show a less favorable response to glucocorticoids and a higher likelihood of requiring surgical intervention, with a variable clinical course and frequent progression to permanent hypopituitarism [10,67].

### 7.3. Immune Checkpoint Inhibitor (ICI)-Induced Hypophysitis

Immune checkpoint inhibitor (ICI)-induced hypophysitis presents a clinical phenotype that depends largely on the immune mechanism involved and the type of drug used. CTLA-4 inhibitors (such as ipilimumab or tremelimumab) are most often associated with a condition reminiscent of classic primary LH, characterized by inflammatory infiltration of the pituitary gland, increased glandular size, and involvement of multiple pituitary axes [24,28,34]. This pattern explains the higher frequency of headache and, in some cases, the appearance of mass effect manifestations. In contrast, PD-1/PD-L1 inhibitors (such as nivolumab or pembrolizumab) tend to produce a different phenotype, in which secondary adrenal insufficiency due to ACTH deficiency predominates, often in isolation and with little or no structural abnormalities on pituitary MRI [44,45]. When combined treatment with CTLA-4 and PD-1 inhibitors is used, the intensity of the immune response increases, which clinically translates into a higher probability of multiple hormonal deficits and radiological findings compatible with hypophysitis, including increased pituitary volume and stalk thickening, as well as an earlier onset of the disease [7,8,33,70,71]. The main clinical, hormonal, and radiological features of pituitary involvement according to the class of immune checkpoint inhibitor are summarized in Table 4.

In patients treated with CTLA-4 inhibitors, hypophysitis usually appears relatively early, typically between 8 and 12 weeks after the start of treatment, or after the third dose in regimens administered every three weeks. In summary of clinical series, the median time to diagnosis is around 10 weeks from the start of treatment. From a clinical point of view, the combination of headache and asthenia is the most characteristic presentation. However, the condition may be accompanied by nonspecific symptoms that often overlap with those derived from the neoplasm itself or from other cancer treatments, such as anorexia, nausea or vomiting, weight loss, dizziness, or decreased libido. Hypotension is less common, but when it occurs, it should raise suspicion of secondary adrenal insufficiency. In terms of hormonal profile, these patients often present with multiple pituitary deficiencies, with the most common being secondary adrenal insufficiency and central hypothyroidism. Hypogonadotropic hypogonadism, hypoprolactinemia, and, with less immediate clinical relevance, growth hormone deficiency may also be observed. Imaging tests often show diffuse enlargement of the pituitary gland, sometimes accompanied by thickening of the pituitary stalk. Visual or field-of-vision abnormalities are rare and are usually limited to cases with significant glandular enlargement. From a semiological point of view, hyponatremia is a relatively common finding in ICI-associated hypophysitis, reported in a significant percentage of patients in various clinical series, which should prompt evaluation of the corticotropic axis upon its appearance [7,8,24,28,34].

Hypophysitis associated with PD-1 or PD-L1 inhibitors presents a distinct clinical pattern. Onset is usually later and variable, with a median time of approximately 27–28 weeks after the start of treatment, although cases have been reported with onset ranging from the first week to several years after the start of immunotherapy, and even after discontinuation of treatment, especially with PD-1 inhibitors. Clinically, the presentation is usually dominated by symptoms derived from secondary adrenal insufficiency, including severe fatigue, myalgia or arthralgia, and anorexia or decreased appetite. Headache and mass effect manifestations are much less common than in CTLA-4-associated hypophysitis. From an endocrinological point of view, isolated ACTH deficiency predominates, or is clearly dominant over other pituitary axes. In retrospective multicenter series cited in recent reviews, most patients were diagnosed based on secondary adrenal insufficiency, while deficiencies in other axes—such as gonadal, growth hormone, or prolactin—were rare. Imaging tests show a lower frequency of pituitary enlargement, with MRI scans typically being normal or showing subtle changes. From a clinical standpoint, the onset of hyponatremia, hypotension, or hypoglycemia in patients undergoing ICI treatment should raise suspicion of secondary adrenal insufficiency, even when the clinical picture is nonspecific [7,8,44,45].

Combination therapy with CTLA-4 and PD-1 inhibitors is associated with a higher incidence and severity of pituitary involvement. Onset is usually early, similar to that observed with CTLA-4 inhibitors in monotherapy, with a median time of approximately 10 weeks from the start of treatment [8]. In prospective studies, the onset of hypophysitis has been reported significantly earlier with combination therapy than with anti-PD-1 monotherapy (median approximately 88 vs. 168 days) [71]. From a clinical and endocrinological point of view, these patients most often present with a classic hypophysitis phenotype, with multiple hormonal deficits and a higher probability of pituitary enlargement [7]. In a prospective study that included patients treated with a combination of ICIs versus monotherapy with PD-1 inhibitors, pituitary dysfunction was significantly more frequent in the combination group (21.6% vs. 3.3%). Similarly, multiple hormonal deficiencies (12.2% vs. 0.3%) and increased pituitary volume on MRI (37.5% vs. 0%) were significantly more common in patients treated with combination therapy. This prospective study included 74 patients receiving combined CTLA-4 and PD-1 blockade and 748 treated with anti-PD-1 monotherapy, with systematic endocrine and radiological follow-up, allowing a more accurate estimation of the incidence and phenotype of pituitary dysfunction. Importantly, ACTH deficiency was present in all cases of pituitary dysfunction, highlighting the particular vulnerability of the corticotropic axis in ICI-induced hypophysitis. In addition, deficiencies of other anterior pituitary axes—particularly gonadotropins and TSH—were significantly more frequent in patients receiving combination therapy, supporting the concept that dual checkpoint blockade induces a broader inflammatory involvement of the pituitary gland [71].

### 7.4. IgG4-Related Hypophysitis

IgG4-related hypophysitis represents a rare form of immune-mediated hypophysitis that belongs to the spectrum of systemic IgG4-related disease and typically affects middle-aged to elderly men, although cases in younger individuals and women have also been reported [10,25,26,72]. Histopathologically, it is characterized by dense infiltration of IgG4-positive plasma cells and storiform fibrosis within the pituitary gland, often accompanied by varying degrees of lymphoplasmacytic inflammation [10,26].

Clinically, the disorder most commonly presents with symptoms related to pituitary hormonal dysfunction together with variable local manifestations. The most frequent symptoms include headache, fatigue, visual disturbances related to sellar mass effect, and constitutional manifestations such as fever, weight loss, anorexia, lethargy, or somnolence, reflecting the systemic inflammatory nature of IgG4-related disease [26,46]. The disorder most commonly presents with multiple anterior pituitary hormone deficiencies, frequently progressing to panhypopituitarism. Among these, ACTH deficiency is of particular clinical relevance due to the risk of life-threatening secondary adrenal insufficiency, while TSH deficiency is also common and requires appropriate thyroid hormone replacement [46]. In contrast to primary LH, involvement of the posterior pituitary and infundibulum is relatively frequent, and AVP-D—manifesting as polyuria and polydipsia—occurs in a substantial proportion of patients [25,26,46]. Symptoms related to sellar mass effect, such as headache or visual impairment, may occur but are generally less prominent than in inflammatory hypophysitis associated with CTLA-4 blockade [10,19]. An important diagnostic feature is the frequent coexistence of extracranial manifestations of IgG4-related disease, including autoimmune pancreatitis, salivary or lacrimal gland enlargement, retroperitoneal fibrosis, or lymphadenopathy, which may provide a critical clue to the systemic nature of the disorder [1,2,3].

### 7.5. Paraneoplastic Immune-Mediated Hypophysitis

Paraneoplastic immune-mediated hypophysitis is an exceptionally rare form of immune-mediated hypophysitis that develops as part of a paraneoplastic immune response triggered by tumors expressing pituitary antigens, leading to loss of immunological tolerance and immune-mediated pituitary damage. The best-characterized entity is anti-PIT-1 hypophysitis, in which cytotoxic T lymphocytes target pituitary cells expressing the transcription factor PIT-1, typically in association with thymoma or other malignancies [27,29,36]. Clinically, this condition is characterized by selective deficiencies of PIT-1–dependent anterior pituitary hormones—growth hormone (GH), prolactin (PRL), and thyroid-stimulating hormone (TSH)—while other pituitary axes are often preserved [27,36]. Symptoms usually reflect these hormonal deficits and may include fatigue, hypothyroidism, and manifestations of GH deficiency. Structural pituitary enlargement and mass effect symptoms are generally minimal or absent, which distinguishes this entity from other inflammatory forms of hypophysitis [36]. Rarely, other paraneoplastic mechanisms may involve additional pituitary antigens—such as POMC—resulting in selective ACTH deficiency in association with neuroendocrine tumors [29].

## 8. Radiological Features

Magnetic resonance imaging (MRI) with gadolinium contrast is the imaging modality of choice for the evaluation of immune-mediated hypophysitis and plays a key role in the differential diagnosis with other sellar lesions. Although imaging findings share some common inflammatory features, the radiological pattern varies according to the underlying etiology.

**Figure 3:**
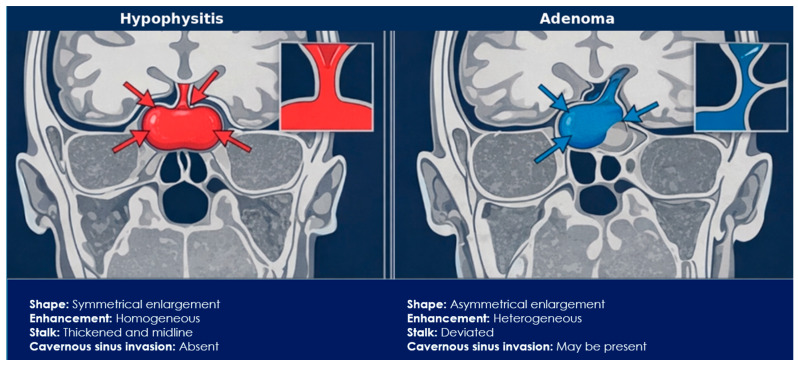
Coronal schematic MRI showing key distinguishing features. Hypophysitis (**left**) demonstrates symmetrical enlargement, homogeneous enhancement, and midline stalk thickening without cavernous sinus invasion, whereas adenomas (**right**) show asymmetrical growth, heterogeneous enhancement, stalk deviation, and possible cavernous sinus invasion.

Primary LH typically shows radiological features consistent with inflammatory enlargement of the pituitary gland. The most characteristic findings include symmetrical pituitary enlargement, homogeneous contrast enhancement, and thickening of the pituitary stalk, usually without lateral deviation [32,57,73,74]. The gland often demonstrates a convex superior margin with mild suprasellar extension, which may mimic a pituitary macroadenoma. However, unlike adenomas, the lesion usually presents uniform enhancement, absence of cavernous sinus invasion, and preservation of the sellar floor [73,74]. These imaging characteristics are particularly useful for differentiating inflammatory hypophysitis from pituitary adenomas in the appropriate clinical context, as illustrated in Figure 3.

When the posterior pituitary is involved, loss of the normal T1 hyperintense posterior pituitary signal (“posterior pituitary bright spot”) may be observed, correlating with AVP-D [74,75]. In the chronic phase, pituitary enlargement may regress spontaneously or after glucocorticoid therapy, occasionally evolving toward pituitary atrophy or empty sella [32,61].

Radiological findings in ICI-related hypophysitis depend largely on the class of immunotherapy. Patients treated with CTLA-4 inhibitors (e.g., ipilimumab) frequently exhibit moderate diffuse pituitary enlargement with homogeneous enhancement and occasional stalk thickening, resembling the inflammatory pattern seen in primary LH [24,28,76,77]. The enlargement is usually transient and may resolve within weeks or months [76]. In contrast, hypophysitis associated with PD-1 or PD-L1 inhibitors (e.g., nivolumab or pembrolizumab) often shows normal or minimally altered pituitary MRI, reflecting the predominance of isolated corticotropic dysfunction without significant structural inflammation [44,45,78,79]. When combined CTLA-4 and PD-1 blockade is administered, radiological abnormalities are more frequent and may include pituitary enlargement and stalk thickening, typically appearing earlier during treatment [80].

IgG4-related hypophysitis commonly demonstrates pituitary enlargement and/or thickening of the pituitary stalk with homogeneous enhancement on MRI. The inflammatory process frequently involves the infundibulum and posterior pituitary, sometimes leading to loss of the posterior pituitary bright spot [46,48]. Compared with LH, the glandular enlargement may be less prominent but is often accompanied by infiltrative changes extending to parasellar structures, occasionally associated with manifestations of systemic IgG4-related disease such as hypertrophic pachymeningitis or orbital pseudotumor [81]. In chronic stages, areas of low T2 signal intensity (“parasellar T2 dark sign”) may reflect collagen deposition and storiform fibrosis [46,81,82].

Radiological abnormalities in paraneoplastic immune-mediated hypophysitis are usually minimal or absent, reflecting selective autoimmune destruction of specific pituitary cell populations rather than diffuse inflammatory enlargement. MRI typically shows a normal-sized or slightly atrophic pituitary gland, although mild glandular enlargement or focal signal heterogeneity may occasionally be observed. Pituitary stalk thickening and mass effect are uncommon, and imaging findings are generally nonspecific [36,52]. Consequently, diagnosis relies primarily on the characteristic endocrine profile and the detection of paraneoplastic autoantibodies rather than on distinctive radiological features.

## 9. Diagnostic Approach and Differential Diagnosis

The diagnostic evaluation of immune-mediated hypophysitis requires a multidisciplinary approach integrating clinical context, endocrine assessment, imaging findings, and, in selected cases, histopathological confirmation. Because the clinical presentation often overlaps with other sellar disorders, early recognition relies on identifying characteristic combinations of hormonal dysfunction, inflammatory imaging patterns, and relevant systemic associations [1,5,10,56].

The initial diagnostic step is a comprehensive hormonal evaluation aimed at identifying anterior pituitary deficiencies and assessing the presence of AVP-D. Particular attention should be given to the corticotropic axis because secondary adrenal insufficiency represents the most clinically significant and potentially life-threatening endocrine deficit. Baseline endocrine assessment should therefore include morning cortisol and ACTH levels, thyroid function tests, gonadotropins and sex steroids, prolactin, insulin-like growth factor-1, and evaluation for AVP-D when clinically suspected [1,10,19,77]. In patients receiving immune checkpoint inhibitors, routine endocrine monitoring during treatment is recommended because hypophysitis may initially present with subtle or nonspecific symptoms [19].

Magnetic resonance imaging with gadolinium contrast is the imaging modality of choice and provides key information for differentiating inflammatory hypophysitis from other sellar masses. Symmetrical pituitary enlargement, homogeneous enhancement, and stalk thickening support an inflammatory process, whereas asymmetric lesions, heterogeneous enhancement, cavernous sinus invasion, or erosion of the sellar floor are more suggestive of pituitary adenoma or other neoplastic conditions. Nevertheless, imaging findings may be normal in certain forms, particularly PD-1/PD-L1 inhibitor-associated hypophysitis, highlighting the importance of integrating radiological and endocrine data [10,19,80]

The differential diagnosis includes pituitary adenomas, metastases, germ cell tumors, Langerhans cell histiocytosis, sarcoidosis, tuberculosis, and other infiltrative or infectious lesions affecting the hypothalamic-pituitary region [60,83,84,85]. Several preoperative scoring systems have been proposed to distinguish immune-mediated hypophysitis from nonfunctioning pituitary adenomas (Table 5). The Gutenberg radiologic score, based on MRI features such as lesion symmetry, homogeneous enhancement, stalk thickening, loss of the posterior pituitary bright spot, and pregnancy association, showed a diagnostic accuracy exceeding 95% in the original cohort [86]. Wright et al. later proposed a simplified clinicoradiologic score including AVP-D, absence of cavernous sinus invasion, infundibular thickening, and absence of visual symptoms; a score ≥3 strongly suggests hypophysitis (AUC 0.96; sensitivity 100%, specificity 75%) [85]. Additional multiparametric models combining clinical, endocrine, and imaging features have also been explored, with higher cumulative scores favoring inflammatory hypophysitis rather than adenoma [87]. Although these tools can improve diagnostic confidence, they should be interpreted in conjunction with the overall clinical context, and histopathological confirmation remains the definitive diagnostic standard in cases of persistent uncertainty.

Systemic clinical features may provide important diagnostic clues: pregnancy or postpartum status favors LH; exposure to immune checkpoint inhibitors suggests treatment-related hypophysitis; multisystem fibroinflammatory manifestations raise suspicion for IgG4-related disease; and the presence of malignancy with selective pituitary hormone deficiencies may indicate paraneoplastic immune-mediated hypophysitis [1,10,19,42].

Pituitary biopsy may be required in selected cases with diagnostic uncertainty, particularly when imaging and clinical findings are inconclusive or when alternative diagnoses such as neoplasms or infections cannot be excluded. However, this procedure is not without risk and may be associated with complications, including cerebrospinal fluid leakage, infection, hemorrhage, and potential worsening of pituitary dysfunction. Therefore, biopsy should be reserved for carefully selected cases and performed by experienced neurosurgeons in specialized centers [19,42,80].

## 10. Management and Therapeutic Strategies

The management of immune-mediated hypophysitis is primarily guided by the severity of endocrine dysfunction, the presence of mass effect, and the underlying etiology (Table 6). In most patients, treatment focuses on prompt recognition and replacement of pituitary hormone deficiencies, particularly secondary adrenal insufficiency, which represents the most urgent and potentially life-threatening complication. Glucocorticoid replacement should therefore be initiated promptly in patients with suspected or confirmed corticotropic deficiency, followed by appropriate substitution of other hormonal axes such as thyroid, gonadal, or growth hormone deficiencies when indicated [1,10,19].

Beyond hormone replacement, the role of anti-inflammatory or immunosuppressive therapy depends on the subtype of immune-mediated hypophysitis and the clinical context. High-dose glucocorticoids are commonly used in patients with significant pituitary enlargement or compressive symptoms, including severe headache or visual disturbances. Corticosteroid therapy may lead to rapid reduction in gland size and improvement of inflammatory symptoms; however, recovery of pituitary function is variable and many patients develop permanent hormonal deficits despite radiological improvement [1,69]. In milder cases without mass effect, conservative management with hormone replacement and close clinical and radiological follow-up is often sufficient.

Accordingly, a pragmatic, severity-based management algorithm can be applied in clinical practice [5]. Patients presenting with compressive symptoms or chiasmatic involvement require prompt administration of high-dose intravenous glucocorticoids followed by early reassessment, with surgical decompression reserved for cases of clinical deterioration or visual compromise. In patients with significant endocrine dysfunction without severe mass effect, oral glucocorticoid therapy with gradual tapering is generally recommended, with close clinical and radiological monitoring. Conversely, in mild or paucisymptomatic cases, conservative management based on hormone replacement and observation is usually appropriate. Long-term follow-up with periodic reassessment is essential in all cases to detect disease progression, relapse, or persistent endocrine deficits.

The therapeutic approach also varies according to the specific subtype of immune-mediated hypophysitis. In immune checkpoint inhibitor (ICI)-induced hypophysitis, management generally consists of hormone replacement rather than aggressive immunosuppression, as pituitary inflammation often stabilizes after treatment initiation. High-dose glucocorticoids are usually reserved for patients with severe mass effect or neurologic symptoms. Importantly, immunotherapy can often be continued once endocrine deficiencies are appropriately managed, particularly in cases presenting with isolated ACTH deficiency [19,79].

In IgG4-related hypophysitis, systemic glucocorticoid therapy represents the first-line treatment and typically results in rapid clinical and radiological improvement. Treatment usually begins with prednisone or prednisolone at doses of approximately 0.6–1.0 mg/kg/day followed by gradual tapering according to clinical and radiological response, and the rapid response to corticosteroids may itself support the diagnosis. In patients with recurrent or refractory disease, additional immunosuppressive agents such as azathioprine, mycophenolate mofetil, methotrexate or B-cell-directed therapies such as rituximab may be considered, particularly in cases with multisystem involvement, severe disease, or glucocorticoid intolerance [47,48,88,89]. In contrast, paraneoplastic immune-mediated hypophysitis requires treatment of the underlying malignancy, while endocrine deficits are managed with hormone replacement [27].

Radiotherapy may be considered as a therapeutic option in selected cases of hypophysitis, particularly in patients with refractory disease, relapsing inflammation, or intolerance to glucocorticoids and immunosuppressive therapies. Although evidence is limited and mainly derived from small case series, radiotherapy may contribute to reducing pituitary mass effects and controlling inflammatory activity. However, its use remains uncommon and should be considered on an individual basis, given the potential risk of long-term hypopituitarism and other radiation-related adverse effects. Therefore, radiotherapy is generally reserved for carefully selected patients and should be delivered in specialized centers [90,91].

Surgical intervention is rarely required and is generally reserved for cases with progressive visual compromise, diagnostic uncertainty, or failure of conservative management [92]. Long-term follow-up is essential, as many patients develop persistent hypopituitarism and require lifelong endocrine monitoring and replacement therapy [1,10,19,69].

## 11. Prognosis and Long-Term Outcomes

The prognosis of immune-mediated hypophysitis is generally favorable in terms of survival, but long-term endocrine outcomes vary considerably depending on the underlying subtype and the extent of pituitary damage. In many patients, particularly those with lymphocytic or immune checkpoint inhibitor (ICI)-induced hypophysitis, persistent hypopituitarism is common despite resolution of pituitary enlargement on imaging. Corticotropic deficiency is the most frequent permanent deficit, whereas recovery of other hormonal axes may occur in a subset of patients over time. In ICI-related hypophysitis, long-term glucocorticoid replacement is often required because ACTH deficiency rarely resolves. In contrast, IgG4-related hypophysitis may show substantial clinical and radiological improvement with glucocorticoid therapy, although relapses and residual endocrine deficits can occur. Overall, these patients require prolonged endocrinological follow-up, as delayed hormonal recovery or progression to permanent pituitary insufficiency may develop even after apparent clinical stabilization [1,6,10,24,48,61,69,75,93].

## 12. Future Directions and Biomarkers

Future research in immune-mediated hypophysitis aims to improve early diagnosis and risk stratification through the identification of reliable biomarkers. Circulating anti-pituitary and anti-hypothalamic antibodies, although not yet standardized, may support the diagnosis of autoimmune pituitary involvement and help identify patients at risk of disease development or recurrence [22,52,94,95]. In ICI-induced hypophysitis, treatment-induced anti-pituitary antibodies and specific HLA haplotypes (e.g., HLA-DR15, HLA-Cw12, HLA-B52) have been proposed as potential susceptibility markers [43,96,97,98]. Emerging autoantibodies such as anti-GNAL (guanine nucleotide-binding protein G(olf) subunit alpha) and anti-ITM2B (integral membrane protein 2B) have also been associated with immune-related endocrine toxicity [99,100].

Advances in immunophenotyping and cytokine profiling, including evidence of Th17-mediated immune responses, may further clarify disease mechanisms and support biomarker development [1,5,10,21]. In addition, novel imaging approaches such as 18F-FDG PET/CT may help detect early pituitary inflammation in patients receiving immunotherapy [76,93,101,102,103]. Despite these advances, non-invasive biomarkers remain investigational, and further controlled studies are required to validate their diagnostic and prognostic utility in immune-mediated hypophysitis [1,10,93].

## 13. Conclusions

Immune-mediated hypophysitis encompasses a heterogeneous spectrum of inflammatory pituitary disorders with distinct etiologies, including primary LH, ICI-induced hypophysitis, IgG4-related disease, and paraneoplastic autoimmune forms. Despite shared immune mechanisms, these entities differ substantially in clinical presentation, radiological features, and therapeutic strategies, underscoring the need for an etiology-oriented approach.

Early recognition is critical, as hypopituitarism—particularly ACTH deficiency—represents the most frequent and potentially life-threatening complication. Diagnosis requires integration of clinical, endocrine, and radiological findings, with clinicoradiological scoring systems providing additional support in selected cases.

Management is primarily based on prompt hormone replacement, complemented by targeted anti-inflammatory or immunosuppressive therapies depending on disease severity and subtype. However, long-term endocrine sequelae remain common, highlighting the need for prolonged follow-up.

Future advances in immunopathogenesis and biomarker discovery are expected to improve early diagnosis, refine risk stratification, and enable more personalized therapeutic strategies in patients with immune-mediated hypophysitis.

## Figures and Tables

**Table 1:** The pituitary gland within the neuroendocrine–immune network.

Functional Axis	Core Component	Key Mechanism & Indicators	References
1. Inputs (The Sensor)	Systemic immune signals	Detection and response to pro-inflammatory cytokines (IL-1β, IL-6, TNF-α) by adenohypophyseal cells; corticotropes show high sensitivity to inflammatory stress.	[12,14]
	Neural & vascular access	Hypothalamic inputs and fenestrated vasculature increase exposure to circulating mediators and facilitate immune cell trafficking, contributing to inflammatory susceptibility.	[11,15]
2. Processing & Integration	Intra-pituitary immune microenvironment	Presence of resident immunity (tissue macrophages, dendritic cells) and local cytokine/chemokine production within the gland.	[13,16]
	Cellular sensitivity	Preferential corticotrope responsiveness with ACTH upregulation during inflammatory stress (HPA axis integration).	[12,14,17]
	Local recruitment	Chemokine expression and transcriptomic programs support pituitary–immune crosstalk and immune cell recruitment.	[18,19]
3. Outputs (The Response)	HPA axis activation	ACTH → cortisol with pleiotropic anti-inflammatory effects (cytokine suppression and immune cell modulation).	[12,14]
	Immunomodulators	Release of POMC-derived peptides with direct immunomodulatory activity during immune stress.	[19,20]
4. Pathophysiology (The Target)	Autoimmune vulnerability	Pituitary as an autoimmune target: lymphocytic infiltration, Th17/IL-17A polarization, and circulating anti-pituitary antibodies.	[10,21,22]
	Induced autoimmunity (ICIs)	Anti-CTLA-4 therapy (ipilimumab): pituitary CTLA-4 expression → local complement activation (type II hypersensitivity); autoantibodies/HLA as risk biomarkers.	[23,24]
	Systemic & paraneoplastic forms	IgG4-related hypophysitis: dense IgG4 + plasma cell infiltration and fibrosis; paraneoplastic autoimmune hypophysitis linked to antigenic mimicry.	[25,26,27]

Abbreviations: ACTH, adrenocorticotropic hormone; CTLA-4, cytotoxic T-lymphocyte–associated antigen 4; HPA, hypothalamic–pituitary–adrenal axis; IgG4, immunoglobulin G4.

**Table 3:** Etiopathogenic mechanisms of immune-mediated hypophysitis according to subtype.

Subtype	Main Immunological Trigger	Dominant Immune Mechanisms	Histopathological Features	Key Pathogenic Consequences	References
**Lymphocytic hypophysitis (LH)**	Primary autoimmune process influenced by genetic and hormonal susceptibility	Predominant **CD4^+^ T-cell infiltration** with Th1/Th17 immune profile; participation of B cells and plasma cells; presence of anti-pituitary autoantibodies	Lymphoplasmacytic inflammatory infiltrates within the pituitary gland	Pituitary enlargement and progressive destruction of endocrine cells that may evolve toward fibrosis and permanent hypopituitarism	[1,21,22,41]
**ICI-induced hypophysitis**	Pharmacological immune activation induced by immune checkpoint inhibitors	Loss of peripheral tolerance with expansion of autoreactive T cells; in anti-CTLA-4 therapy, antibody binding to pituitary CTLA-4 with **complement-mediated cytotoxicity**; induction or amplification of anti-pituitary autoantibodies	Acute inflammatory infiltrate; typically less organized than primary LH and less prone to fibrosis	Inflammatory pituitary enlargement and multiple hormonal deficits (anti-CTLA-4) or selective corticotroph dysfunction (anti-PD-1/PD-L1)	[23,24,33,42,43,44]
**IgG4-related hypophysitis**	Immune dysregulation within systemic **IgG4-related disease**	Expansion of IgG4-secreting plasma cells driven by B cells and Th2/Treg immune responses; cytokines such as IL-4, IL-10 and IL-13 promote IgG4 class switching	Dense lymphoplasmacytic infiltrate rich in IgG4^+^ plasma cells with **storiform fibrosis** and occasionally obliterative phlebitis	Chronic fibroinflammatory remodeling leading to pituitary enlargement and progressive endocrine dysfunction	[25,26,35,40]
**Paraneoplastic autoimmune hypophysitis**	Immune response against tumor antigens that cross-react with pituitary proteins (**antigenic mimicry**)	Activation of autoreactive CD4^+^ and CD8^+^ T cells triggered by tumor antigen presentation; production of circulating autoantibodies against pituitary antigens	Immune-mediated infiltration with selective destruction of hormone-producing cell populations	Selective pituitary hormone deficiencies; prototypical example is **anti-PIT-1 hypophysitis**, affecting somatotrophs, lactotrophs, and thyrotrophs	[27,29,36]

Abbreviations: ACTH, adrenocorticotropic hormone; CTLA-4, cytotoxic T-lymphocyte-associated antigen 4; ICI, immune checkpoint inhibitor; IgG4, immunoglobulin G4; IL, interleukin; LH, lymphocytic hypophysitis; PD-1, programmed cell death protein 1; PD-L1, programmed death ligand 1; PIT-1, pituitary-specific transcription factor-1; Treg, regulatory T cell.

**Table 4:** Clinical characteristics of pituitary involvement according to the class of immune checkpoint inhibitor therapy.

Characteristic	Anti-CTLA-4 (Ipilimumab, Tremelimumab)	Anti-PD-1/PD-L1 (Nivolumab, Pembrolizumab, Atezolizumab, etc.)	Combination CTLA-4 + PD-1	References
**Predominant phenotype**	Inflammatory hypophysitis resembling primary lymphocytic hypophysitis, with pituitary enlargement and involvement of multiple pituitary axes	Predominantly isolated or dominant ACTH deficiency with minimal inflammatory enlargement	More severe inflammatory phenotype with a higher frequency of multiple hormonal deficiencies	[7,8,24,25]
**Typical onset**	Early onset, usually within 2–3 months after treatment initiation	Later and more variable onset, sometimes months after treatment initiation or even after discontinuation	Early onset, generally similar to CTLA-4 inhibitors and earlier than PD-1 monotherapy	[8,28,34,71]
**Dominant clinical symptoms**	Headache and asthenia are common; nonspecific symptoms such as nausea, anorexia, dizziness, or weight loss may occur	Severe fatigue and symptoms related to secondary adrenal insufficiency; headache less frequent	Similar to CTLA-4 inhibitors but with a higher burden of hypopituitarism-related symptoms	[7,24,28]
**Most affected pituitary axis**	Corticotropic axis (ACTH) frequently affected together with other axes	Corticotropic axis predominantly affected, often in isolation	Corticotropic deficiency present in most cases	[7,24,44,71]
**Other hormonal deficiencies**	Frequent: central hypothyroidism and hypogonadotropic hypogonadism; occasionally prolactin or GH deficiency	Rare involvement of other axes	Higher prevalence of multiple deficiencies, particularly gonadotropin and TSH deficits	[7,8,25,71]
**Pituitary MRI findings**	Diffuse pituitary enlargement often present, sometimes with stalk thickening	MRI usually normal or with subtle abnormalities	Higher probability of pituitary enlargement	[7,8,25]
**Mass-effect manifestations**	Rare but possible when gland enlargement is significant	Very rare	Uncommon but more likely than with PD-1 monotherapy	[25,28]
**Biochemical clues**	Hyponatremia relatively frequent due to secondary adrenal insufficiency	Hyponatremia, hypotension, or hypoglycemia may be presenting clues	Similar findings but with higher probability of multiple hormonal deficits	[7,8,24]
**Incidence of pituitary dysfunction**	Intermediate incidence compared with other ICI classes	Low incidence	Highest incidence among ICI regimens	[8,33,71]

Abbreviations: ACTH, adrenocorticotropic hormone; CTLA-4, cytotoxic T-lymphocyte-associated antigen 4; GH, growth hormone; ICI, immune checkpoint inhibitor; MRI, magnetic resonance imaging; PD-1, programmed cell death protein 1; PD-L1, programmed death ligand 1; TSH, thyroid-stimulating hormone.

**Table 5:** Preoperative scoring systems proposed to differentiate autoimmune hypophysitis from pituitary adenomas.

Scoring System	Main Variables Included	Diagnostic Performance	Clinical Utility	Author, Year/Key Reference
**Gutenberg radiologic score**	Symmetrical pituitary enlargement, homogeneous contrast enhancement, pituitary stalk thickening, loss of posterior pituitary bright spot, association with pregnancy/postpartum status	Diagnostic accuracy reported > 90% in the original cohort	One of the earliest and most widely cited radiologic scores; useful when inflammatory imaging features predominate	Gutenberg et al., 2009 [86]
**Wright clinicoradiologic score**	Diabetes insipidus, absence of cavernous sinus invasion, pituitary stalk thickening, absence of visual symptoms	Score ≥ 3: sensitivity 100%, specificity 75%, AUC 0.96	Simple clinical-radiologic tool to estimate the probability of hypophysitis before surgery	Wright et al., 2022 [85]
**Multiparametric predictive models**	Combination of clinical variables (sex, pregnancy status), endocrine profile, MRI features (symmetry, stalk involvement, enhancement pattern)	AUC ≈ 0.90 in exploratory cohorts	Integrates clinical, hormonal, and imaging variables; may reduce unnecessary surgical exploration	Taieb et al., 2025 [87]

**Table 6:** Therapeutic strategies according to subtype of immune-mediated hypophysitis.

Subtype	First-Line Treatment	Role of Glucocorticoids	Additional Therapies	References
**LH**	Hormone replacement	For mass effect or severe inflammation	Rarely immunosuppressants	[1,10]
**ICI-induced hypophysitis**	Hormone replacement (ACTH deficiency)	Reserved for severe symptoms	Usually not required	[19,79]
**IgG4-related hypophysitis**	Systemic glucocorticoids	First-line therapy	Immunosuppressants (azathioprine, mycophenolate, methotrexate) or rituximab in refractory cases	[47,48,88,89]
**Paraneoplastic autoimmune hypophysitis**	Hormone replacement	Limited role	Treat underlying malignancy	[27]

Abbreviations: ICI, immune checkpoint inhibitor; ACTH, adrenocorticotropic hormone; LH, lymphocytic hypophysitis.

## Data Availability

No new data were created or analyzed in this study.
